# A Review of Oxytocin and Arginine-Vasopressin Receptors and Their Modulation of Autism Spectrum Disorder

**DOI:** 10.3389/fnmol.2018.00027

**Published:** 2018-02-13

**Authors:** Ilaria Cataldo, Atiqah Azhari, Gianluca Esposito

**Affiliations:** ^1^Department of Psychology and Cognitive Science, University of Trento, Rovereto, Italy; ^2^Mobile and Social Computing Lab, Fondazione Bruno Kessler, Trento, Italy; ^3^Division of Psychology, School of Social Sciences, Nanyang Technological University, Singapore, Singapore

**Keywords:** *OXTr*, *AVPr*, rs53576, rs2254298, rs2268493, Autism Spectrum Disorder (ASD)

## Abstract

Oxytocin (*OXT*) and arginine-vasopressin (*AVP*) play a key regulatory part in social and affiliative behaviors; two aspects highly compromised in Autism Spectrum Disorder (ASD). Furthermore, variants in the adjacent oxytocin-vasopressin gene regions have been found to be associated with ASD diagnosis and endophenotypes. This review focuses mainly on common *OXTr* single nucleotide polymorphisms (SNPs), *AVPR1a* microsatellites and *AVPR1b* polymorphisms in relation to the development of autism. Although these genes did not surface in genome-wide association studies, evidence supports the hypothesis that these receptors and their polymorphisms are widely involved in the regulation of social behavior, and in modulating neural and physiological pathways contributing to the etiology of ASD. With a specific focus on variants considered to be among the most prevalent in the development of ASD, these issues will be discussed in-depth and suggestions to approach inconsistencies in the present literature will be provided. Translational implications and future directions are deliberated from a short-term and a forward-looking perspective. While the scientific community has made significant progress in enhancing our understanding of ASD, more research is required for the ontology of this disorder to be fully elucidated. By supplementing information related to genetics, highlighting the differences across male and female sexes, this review provides a wider view of the current state of knowledge of *OXTr* and *AVPr* mechanisms of functioning, eventually addressing future research in the identification of further risk factors, to build new strategies for early interventions.

## Introduction

Afflicting individuals across ethnic and socioeconomic divides, autism spectrum disorder (ASD) is a severe neurodevelopmental condition known to first emerge in individuals before the age of three (Fombonne, 2009; American Psychiatric Association, 2013). Occurring 4 times more in boys than girls (Baron-Cohen et al., 2005; Steyaert and De la Marche, 2008; Lintas and Persico, 2009), this severe developmental disorder is characterized by three diagnostic symptom domains: impairments in social interaction, verbal and non-verbal communication, and restricted, repetitive patterns of behavior, interests or activities (American Psychiatric Association, 2013). The autistic phenotype leaves individuals less adept at normal social interaction (Kanner, 1968; Clifford et al., 2007), expressive language, non-verbal communication (e.g., maintaining eye contact) (Lord et al., 2000; Hill and Frith, 2003; Groen et al., 2008), peer relationships (Kanner, 1968; Clifford et al., 2007) and flexibility in behavioral patterns (Szatmari et al., 2006). Beyond these two broad categories, a diverse constellation of symptoms frequently co-occur with ASD diagnosis. These include hyperactivity (Volkmar et al., 1999; Simonoff et al., 2008), sensory dysfunction (Baranek et al., 2007; Tommerdahl et al., 2007; Cascio et al., 2008; Coskun et al., 2009; Boyd et al., 2010; Kwakye et al., 2011) and intellectual disability (Chakrabarti and Fombonne, 2005). With an estimated heritability of around 64–91% in twin studies (Tick et al., 2016) and 31–71% in whole-genome genotyping studies (Gaugler et al., 2014), compelling evidence suggests that the etiology of ASD bears a strong genetic influence (Szatmari et al., 2006; Abrahams and Geschwind, 2008). An array of approaches, including genome-wide association studies, genetic linkage and expression analysis have been utilized to identify individual genes contributing to ASD (Veenstra-VanderWeele and Cook, 2004; Vorstman et al., 2006; Freitag, 2007; Gupta and State, 2007; Abrahams and Geschwind, 2008; Wang et al., 2009). Kumar and Christian (2009) have also reported that numerous types of genetic disorders, from mutations in single nucleotide bases to irregularities in chromosomal structures and numbers, can lead to the development of an autistic phenotype (Buxbaum, 2009).

In addition to complex genetic abnormalities, the quest to uncovering the causes of ASD has been rendered more difficult by the contributing role of environmental factors, including toxins, pesticides *in utero* exposure to drugs and maternal infection (Moore et al., 2000; Rasalam et al., 2005; St-Hilaire et al., 2012; Volk et al., 2013; Berko et al., 2014; Sandin et al., 2014; Tordjman et al., 2014). Some of these factors might exert their influence during pregnancy (Depino, 2017; Nicolini and Fahnestock, 2017) while others might occur during critical stages of development. One environmental variable that has been well-established to increase the risk of ASD is maternal infection during early fetal neurodevelopment (Atladóttir et al., 2010; Zerbo et al., 2013, 2015; Lee et al., 2015; Mazina et al., 2015). Given the wide-ranging detrimental effects of prenatal infection (e.g., rubella and cytomegalovirus) on the central nervous system (Johnson, 1994), it is to no surprise that prenatal infection has emerged as a notable environmental risk factor for ASD. While convergent findings on the role of prenatal infection in ASD development are readily available in the existing literature, numerous differences still prevail in pin-pointing the specific causative agent and exact prenatal period involved. For instance, Atladóttir et al. (2010) found that viral infection in the first trimester, and any infection in the second trimester led to increased risk of ASD. Contrary to the agent- and trimester-specific associations highlighted in 2010, Lee et al. (2015) later discovered that infectious agents of any type (i.e., bacterial, viral, other infection), during the entire course of pregnancy, can contribute to ASD development. To elucidate the mechanism of action of these infections, numerous animal model studies have been conducted, revealing that prenatal infection, either through direct exposure of preganant mice to viruses, or via activation of maternal immune response in the absence of an infectious agent, led to atypical behaviors in the resulting pups (Brown and Derkits, 2010; Meyer et al., 2011; Patterson, 2011; Miller V. M. et al., 2013). This suggests that infectious agents, albeit possessing individual characteristics, share common biological pathways that influence the development of ASD. One theory that has been gaining recognition in recent years is the cytokine hypothesis. Cytokines, which are signaling molecules in the immune system, play a critical role in neuronal migration, survival and development (Deverman and Patterson, 2009). Maternal infection is hypothesized to trigger a dysregulation in cytokine levels, which disrupts typical neurodevelopment and contributes to ASD (Ashwood et al., 2006, 2011). Beyond the fetal period, environmental factors have also been found to induce epigenetic changes. For instance, intense social experiences in sensitive life stages can have enduring influences not only on behavior, but also on *AVPR1a* gene expression in the hippocampal region through a change in patterns of methylation (Bodden et al., 2017).

Recent epidemiological studies demonstrate a disturbing upward trend in number of ASD cases, with an estimate of 1 in every 68 children being diagnosed with ASD (NIMH statistics prevalence of autism). Some researchers have proposed this increase to be due to an enhanced awareness of the disorder or a reduced strictness in diagnoses (for example, see Rutter, 2005), while others point to the possibility of a true increase in number of individuals with ASD (Fombonne, 2002; Inglese and Elder, 2009). In light of these prevailing statistics, efforts in the search for the roots of ASD has greatly increased. However, such efforts have generated inconsistent results due to heterogeneity of ASD and the polygenic manner in which various genetic loci contribute to the disorder (Betancur, 2011; Geschwind, 2011). One possible way to parse the heterogeneity of ASD may be through the use of biological biomarkers (Hazlett et al., 2005; Redcay and Courchesne, 2005; Giulivi et al., 2010; Schumann et al., 2010; Courchesne et al., 2011a,b; Rossignol and Frye, 2012). In this review, our focus will be directed at the genetic level, systematically examining the role of single nucleotide polymorphisms (SNPs) in oxytocin receptor (*OXTr*) and arginine-vasopressin receptor (*AVPr*) genes in the etiology of ASD. Besides having been established to play an imperative role in social behavior and affiliation (Insel and Shapiro, 1992; Panksepp, 1992; Veenema and Neumann, 2008), *OXTr* and *AVPr* have also been previously associated with ASD (Israel et al., 2008). Aside from their involvement in regulating behaviors, this review focuses on these two receptor classes as they exhibit different protein expression patterns in males and females, an essential biological characteristic that ought to be taken into account while approaching strongly sex-biased disorders such as ASD.

### Genome-wide association studies vs. candidate-gene studies

The latest technological advancements in the genetic field has allowed researchers the opportunity to detect specific gene loci involved in neuropsychiatric and developmental disorders. In the 1980's, researchers began to consider the possibility of a strong genetic component underpinning ASD. Since then, many genes were found to be associated to autistic traits. Genome-wide association studies (GWAS) highlighted the association between autism and genes, from rare ones, such as *PARK2* (Yin et al., 2016), to those which are more common, like *SHANK3* (Nemirovsky et al., 2015; Sanders et al., 2015; Guo et al., 2017), *FOXP1, FHIT*, (The Autism Spectrum Disorders Working Group of The Psychiatric Genomics Consortium, 2017) and independent polymorphisms, like rs57709857-A (The Autism Spectrum Disorders Working Group of The Psychiatric Genomics Consortium, 2017). Although this technique efficiently reveals genes involved in autism within a shorter period of time, findings should be interpreted with caution given the great overlap present between genes involved in ASD and other medical/neuropsychiatric conditions. The current literature presents evidence of shared genetic, biological, and physiological etiology between mental disorders, such as ASD and ADHD (Craig et al., 2015; Panagiotidi et al., 2017), schizophrenia (Ishizuka et al., 2017; Prata et al., 2017), major depressive disorder (Glessner et al., 2017), and medical conditions like epilepsy (for a review see Strasser et al., 2017) and disorders of the immune system (Cieślinska et al., 2017; Li et al., 2017). Aside from these considerations, other limiting aspects of GWAS studies include the need for greater sample sizes and difficulties in replicating studies involving a population with ASD. These complexities may be due to the heterogeneous nature of the disorder, differences among ethnicities, and other environmental factors which render genome-wide studies useful but not resolutive.

Candidate gene studies is an alternative approach that requires large sample sizes to explore haplotypes, single genes, or even a few polymorphisms at a time. Some optimistic developments in research of *OXTr* and *AVPr* receptor classes have been required through the use of candidate gene studies. For instance, this method has elucidated specific associations of *OXTr* and *AVPr* polymorphisms to specific structures of the brain (e.g., amygdala) (Schneider-Hassloff et al., 2016), as well as to downstream physiological pathways such as the hypothalamic-pituitary axis (Auer et al., 2015). Furthermore, this methodology is more applicable to the new criteria of ASD diagnosis found in the latest edition of the Diagnostic and Statistical Manual of Mental Disorders (DSM-5), which regards disorders to exist on a continuous spectrum, ranging from the general population to the severely impaired clinical group.

### ASD in the DSM-5

Autism has been shown to be a heterogeneous and complex condition with multiple etiologies, resulting in a diverse array of clinical presentations across individuals. With revisions in the clustering of symptoms and criteria for diagnosis, DSM-5 has prescribed to the notion of “spectrum,” giving importance not only to singular features but also to the extent to which these symptoms impair daily functioning. Ever since this latest DSM edition has been applied to clinical practices, a decrease in number of ASD diagnoses has been observed (Kulage et al., 2014; Sturmey and Dalfern, 2014; Smith et al., 2015). In distinguishing the degree of severity of ASD symptoms, this paradigm shift has established a continuum of autistic traits that ranges from the mild to the severely impaired. DSM-5 more accurately reflects how social behaviors widely differ from person to person, making it possible for autistic traits to be found in the general population too, especially in the case where individuals share common environmental factors (Skylark and Baron-Cohen, 2017; Suzuki et al., 2017). Recently, it has been observed that ASD and autistic traits also share similar genetic and biological elements (Bralten et al., 2017), but it is necessary to take a look at these information emerging from literature holistically, before speculating the mechanisms underlying the development of ASD. The pursuit of research that investigates common autistic characteristics across both clinical and non-clinical populations might be helpful in developing more streamlined diagnostic practices.

### Lessons from animal models

In the numerous attempts to define the etiology of autism, much progress has been made possible due to research on animal models, which has exponentially increased since the use of the transgenic mouse. To date, this technique has allowed researchers to manipulate genetic information (Halladay et al., 2009), allowing control over genetic factors that contribute to the development of neurodevelopmental disorders, potentially giving rise to new medical treatments (Avraham et al., 2017). Rodent models have been adopted in ASD studies to investigate mechanisms for prevention (Vuillermot et al., 2017), and potential therapeutic treatments (Chadman, 2017; Guoynes et al., 2017), as well as to better understand the involvement of the gut-brain axis (for a review see Nithianantharajah et al., 2017) and the immune system (Schwartzer et al., 2017) in ASD etiology. To illustrate this, we draw specific attention to the pivotal role that animal models have played in elucidating how maternal immune activation, a well-ascertained environmental risk factor, contributes to ASD. Using immune activating agents, such as polyinosinic-polycytidylic acid (PolyIC), these studies induce an activation of the maternal immune system in pregnant rodents (Medzhitov, 2001). Following that, parameters of ASD-like behaviors in the offspring, such as pup ultrasonic vocalizations (Malkova et al., 2012; Hsiao et al., 2013; Schwartzer et al., 2013; Choi et al., 2016) and brain abnormalities, are compared against parallel ASD characteristics in humans (for reviews, see Meyer et al., 2009; Boksa, 2010). Cross-species evaluation has generated interest in examining the evolutionarily conserved effects of maternal immune activation on ASD-like behaviors (Belzung and Lemoine, 2011; Stewart and Kalueff, 2015) in rat and non-human primate models as well. While mice models have been the long-standing preferred species in biomedical research due to the extensive range of possible genetic manipulations, rat models feature a more complex array of brain activities and enriched social behaviors (Yee et al., 2012; Vanderschuren and Trezza, 2013; Ku et al., 2016), that serve as an advantage for studies relating to autism, since this disorder primarily features a deficiency in social cognition (Couture et al., 2010). Despite the numerous advantages that the rodent species has to offer, the social signals (i.e., vocalizations, facial expressions, body gestures) and underlying patterns of brain activity in rodents are limited (Chang et al., 2013). Thus, non-human primate models, such as the rhesus monkey, bridges the link from rodent models to humans (Watson and Platt, 2012; Platt et al., 2016), although such studies may be rife with ethical concerns.

With respect to *OXT* and *AVP* systems, evidence in literature highlights the contribution of these two peptides in social processes (Modi and Young, 2012; Peñagarikano et al., 2015; Zimmermann et al., 2016). Engineering of animal models specific to genes implicated in these systems is crucial in aiding the investigation of ASD etiology from this research front (Hammock and Young, 2006; Murakami et al., 2011; Peñagarikano, 2017). Indeed, OXT and OXTr knockout (KO) mice have been found to exhibit abnormal social behaviors akin to ASD-like traits (Ferguson et al., 2000; Winslow and Insel, 2002) and futher studies have begun to uncover the specific biological pathways involved. For example, Fujiwara et al. (2016) have recently investigated the role of HPC-1/syntax-in1A (STX1A), a N-ethylmaleimide-sensitive fusion attachment protein receptor complex which regulates the synaptic transmission of OXT (Südhof and Rothman, 2009). Employing a social novelty preference test, they found that STX1A KO mice showed unusual social behavior that is thought to be a component of a stereotypic ASD-like behavioral profile (Burket et al., 2011), also displayed by OXTR KO mice (Fujiwara et al., 2010). However, intracerebroventricular administration of oxytocin (OXT) partially rescued this phenotype (Fujiwara et al., 2016). This is a prime depiction of how animal model studies illuminate biological pathways related to the ASD phenotype as well as gives insight into preclinical research options that potentially advances the treatment of this disorder.

### OXTr and AVPR

The core symptoms of autism revolve around deficits in normal social communication (Snow et al., 2009; Sala et al., 2011). Two neuropeptides of interest, oxytocin (*OXT*) and arginine-vasopressin (*AVP*), have been well-documented to mediate social and cognitive processes in clinical and non-clinical settings (Neumann, 2008; Ebstein et al., 2009, 2012), as well as across species (Veenema and Neumann, 2008; Ebstein et al., 2009; Zhang et al., 2017). They have been established to facilitate biologically adaptive social behaviors, such as trust (Kosfeld et al., 2005) positive communication (Ditzen et al., 2009), maternal care (Bielsky et al., 2005; Bosch and Neumann, 2008), and affiliation (Lim et al., 2005; Veenema et al., 2010). Because of their integral function in shaping social conduct, researchers began to postulate the involvement of these neuropeptides in the etiology of ASD (Insel et al., 1999; Hammock and Young, 2006; Watanabe et al., 2017). Indeed, animal model studies have shown that administration of *OXT* and *AVP* were able to rescue autistic traits (Sala et al., 2011) and increase social recognition memory (Bielsky and Young, 2004; Winslow and Insel, 2004), while in humans, intranasal *OXT* has been reported to heighten the understanding of others' mental states (Domes et al., 2007) - a deficit found amongst individuals with ASD (Caronna et al., 2008). As these neuropeptides exert their behavioral effects upon binding to three specific G-protein-coupled (Gimpl and Fahrenholz, 2001; Thibonnier et al., 2002) receptors; *OXTr, AVPR1a*, and *AVPr1b* (Manning et al., 2008), the next advance in this field was made when scientists looked toward *OXTr* (Wu et al., 2005; Jacob et al., 2007; Lerer et al., 2008) and *AVPr* (Kim et al., 2002; Wassink et al., 2004; Yirmiya et al., 2006) as potential candidate genes (for a review, see Zhang et al., 2017).

**Figure 1:**
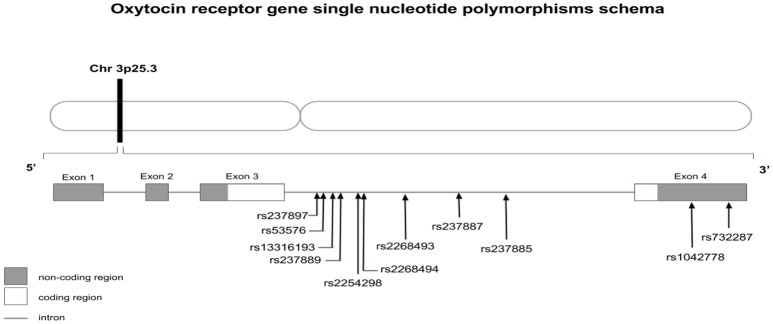
Oxytocin receptor gene schema with location of discussed single nucleotides polymorphisms.

Known for its role in facilitating social behavior, *OXTr* has been implicated in neuropsychiatric disorders, ranging from social anxiety disorders and schizophrenia (Wu et al., 2005; Jacob et al., 2007; Lerer et al., 2008; Yrigollen et al., 2008; Ebstein et al., 2009; Gregory et al., 2009; Liu et al., 2010; Guastella and MacLeod, 2012; Meyer-Lindenberg and Tost, 2012) to autism (Wu et al., 2005; Ylisaukko-oja et al., 2006; Jacob et al., 2007; Lerer et al., 2008; Yrigollen et al., 2008; Gregory et al., 2009; Liu et al., 2010; Campbell et al., 2011). Based on numerous genome-wide association studies, *OXTr* has been proposed to be a potential risk gene in ASD. Supporting this proposition is a 0.7 Mb deletion in 3p25.3, which has been found to occur with autism (see Figure 1). Since *OXTr* was encompassed in the deleted region, such mutations implicating *OXTr* were suggested to be involved in ASD etiology (Wu et al., 2005; Jacob et al., 2007; Lerer et al., 2008). However, this phenomenon appears to be rare as other studies have not reported *OXTr* deletions typifying autism (Sebat et al., 2007; Marshall et al., 2008; Glessner et al., 2009; Sanders et al., 2015). As compared to deletions, the association of *OXTr* SNPs with ASD has been more commonly observed. Several SNPs of *OXTr* have been linked to ASD, including rs237887, rs2268491 and rs225429, rs7632287 (Ylisaukko-oja et al., 2006; Jacob et al., 2007; Lerer et al., 2008; Yrigollen et al., 2008; Ebstein et al., 2009; Liu et al., 2010; Campbell et al., 2011; Walum et al., 2012; LoParo and Waldman, 2015). However, some studies did not relate any association (Tansey et al., 2010; Wermter et al., 2010; Verhallen et al., 2017), while others reported ethnic differences in the association of *OXTr* SNPs with autism (Wu et al., 2005; Jacob et al., 2007; Lerer et al., 2008; Liu et al., 2010).

**Figure 2:**
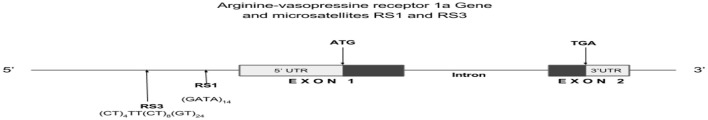
Arginine-vasopressin receptor 1a gene schema with location of microsatellites RS1 and RS3.

In the domain of social behavior, *AVPR1a* (Lim et al., 2005; Veenema et al., 2010) and *AVPR1b* are two integral players from the *AVP* system. Well-known to be located on chromosome 12q14-15, the gene for *AVPR1a* possesses three microsatellites in the 5u flanking region and a fourth one in an intronic region (Thibonnier et al., 2000; Thibonnier, 2004) (see Figure 2). Expression patterns of *AVPR1a* in the brain significantly predict differences in social behavior of voles within and across species (Hammock and Young, 2002, 2006). Of clinical relevance, *AVPR1a* polymorphism has been shown to be involved in social recognition (Bielsky et al., 2005), amygdala activation (Meyer-Lindenberg et al., 2009) and implicated in ASD (Kim et al., 2002; Wassink et al., 2004; Yirmiya et al., 2006). In 2002, Kim et al. (2002) was the first group to document the significant transmission disequilibrium of a microsatellite on *AVPR1a* and autism, before Wassink et al. (2004) and Yirmiya et al. (2006) independently reproduced this finding. (Israel et al., 2008) found that deficits in socialization skills mediated this link. For the past decade, the central wellspring of research in *AVPr* lies in the promoter region of two microsatellites, RS1 and RS3, where length of microsatellites has been found to modulate social behavior in human and lower mammals (Israel et al., 2008; Knafo et al., 2008). This observation was supported by corresponding mRNA analyses, in which longer *AVPR1a* RS3 repeats were found to be associated with higher levels of *AVPR1a* mRNA expression (Knafo et al., 2008). This asserts the functional role of different *AVPR1a* microsatellites in determining social behavior. Of less prominence in the study of autism is the receptor *AVPR1b*, which has been previously linked to aggression, anxiety, pro-sociality and autistic traits (Dempster, 2007; Chakrabarti et al., 2009; Dempster et al., 2009; Zai et al., 2012; Wu et al., 2015). Only recently, Francis et al. (2016) reported a significant association between *AVPR1b* and ASD.

Having chronicled the consensus and contention surrounding *OXTr* and *AVPr* in ASD, we have established the foundation for this review. Thereafter, we will endeavor to highlight specific polymorphisms of *OXTr* and *AVPr* that have been either recently or repeatedly associated with autism. Exploring the breadth of the current literature, we will provide suggestions on how inconsistencies can be approached and reconciled, as well as recommend possible future directions that might advance this field of research further.

## Methods

**Figure 3:**
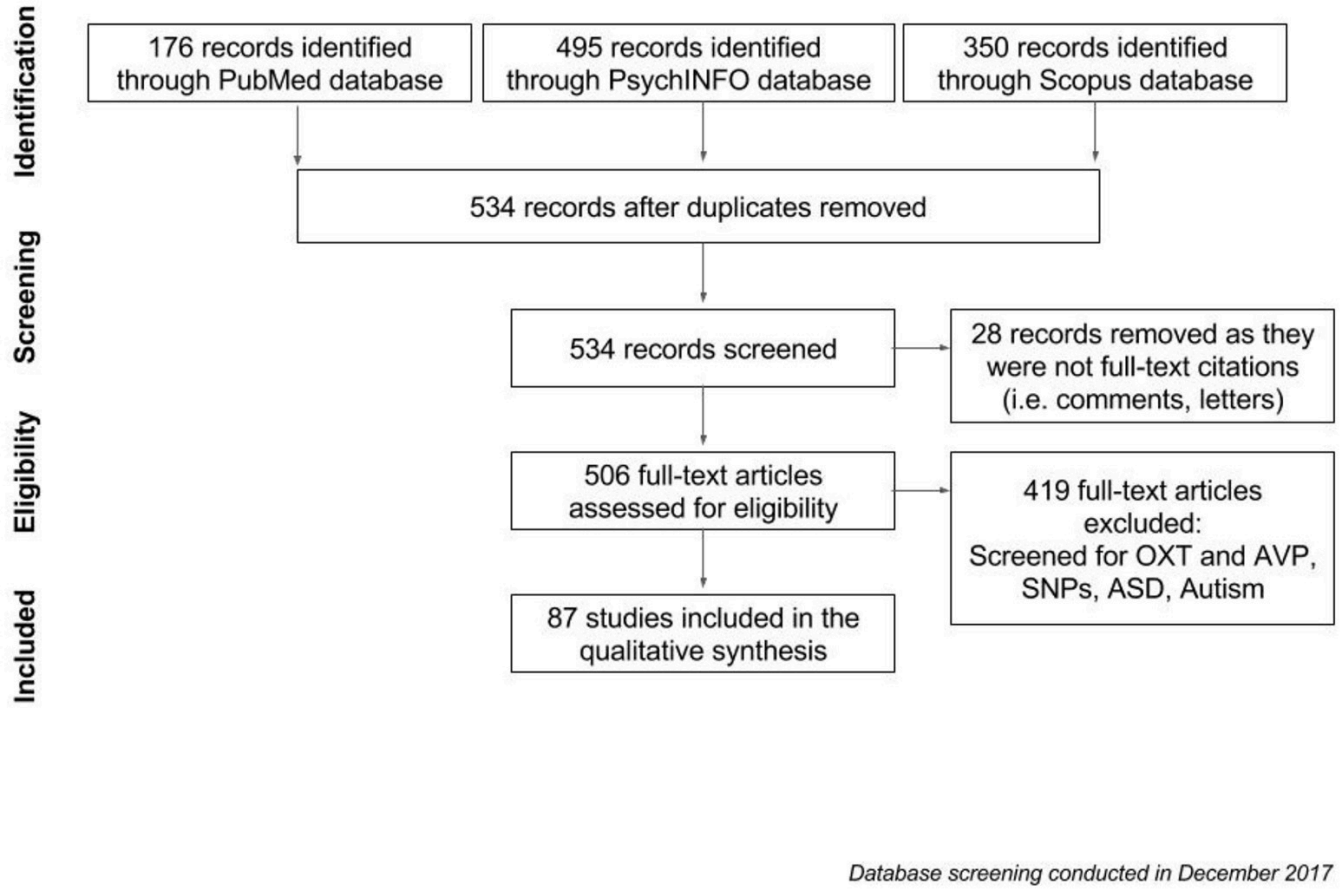
Flowchart of the screening process for articles to be included in qualitative analysis.

Pubmed, PsycINFO and Scopus databases were used to browse for articles on the oxytocin receptor and arginine vasopressin systems. We comparatively analyzed the entire literature up to December 2017, combining different keywords and Boolean operators (see Figure 3). Firstly, we collated all papers that were generated by the term [“*OXTr*” OR “*AVPr*” AND “ASD”], before adding the results of a new search [“oxytocin receptor” OR “arginine vasopressin receptor” AND “autism”]. 176 papers were generated through Pubmed, and 495 and 350 papers were obtained from PsycINFO and Scopus respectively. After duplicates were removed, results from this search amounted to a list of 534 records. Twenty-eight records were removed as they were not full-text citations (i.e., comments, letters), leaving 506 articles to be assessed for eligibility. After filtering for relevant papers based on whether they contained information on “*AVPr*,” “*OXTr*,” and “ASD,” 87 studies remained and were included in the qualitative analysis (see Table 1 for the list of papers included in the review). These papers were sub-categorized into different lists according to fixed criteria: receptor in the text (oxytocin and/or arginine vasopressin) related to the ASD topic, species discussed (human/animal), and SNP involved in the research. With regard to *AVPr*, we further subdivided the papers according to both the specific receptor group and the main polymorphism that has been found to be related to the risk of developing ASD; these are *AVPR1a* (microsatellites RS1 and RS3 mainly) and *AVPR1b* (polymorphisms rs28632197 and rs3536969). As for *OXTr*, we divided the papers based on the SNPs which were investigated (rs53576, rs2254298, rs2268493). The following section will explore, in greater detail, how SNPs of these receptors contribute to the development of autism, with a specific aim of illustrating how this dual modulation underlies autistic traits in clinical and non-clinical populations.

**Table 1:** Articles included in the review, with SNPs involved, sample size, and *p*-values.

**Manuscript**	**Topic**	**Gene/SNP involved**	**Sample size**	***p*-value**
Abrahams and Geschwind, 2008	Neurobiology of ASD	OXTr – AVPR1a	Review article	–
Apicella et al., 2010	Social preferences	OXTr rs75775 OXTr rs2268493 OXTr rs1042778	684 participants	>0.05
Auer et al., 2015	Rejection sensitivity	OXTr rs53576	94 participants	0.036
Avinun et al., 2012	Maternal behavior	AVPR1a RS3	252 dyads (mother – twins)	<0.01
Avinun et al., 2011	Altruistic behavior	AVPR1a RS3	158 twins	0.04
Bakermans-Kranenburg and van Ijzendoorn, 2014	Social behavior	OXTr rs53576 OXTr rs2254298	17557 participants 13452 participants	>0.05 >0.05
Bielsky and Young, 2004	Social recognition	AVPR1a AVPR1b OXTr	Review article	–
Bielsky et al., 2005	Social recognition	AVPR1a	Mice	<0.01
Brüne, 2012	Psychopathology	OXTr rs2254298	Review article	–
Caldwell et al., 2010	Social dominance	AVPR1b	Mice	<0.04
Caldwell et al., 2008	Aggression	AVPR1b	Review article	–
Campbell et al., 2011	Association study	OXTr2268493 OXTr rs1042778 OXTr rs7632287	2333 ASD	0.043 0.037 0.016
Chakrabarti et al., 2009	Autistic traits	OXTr SNPs AVPR1a AVPR1b	349 participants	>0.05 >0.05 >0.05
Chang et al., 2014	Social integration	OXTr rs53576 AVPR1a	>11.000 individuals	0.02 >0.05
Chen et al., 2011	Social support and stress	OXTr rs53576 OXTr rs2268493	194 participants	<0.01 0.00637
Di Napoli et al., 2014	Autistic traits and Asperger	OXTr rs53576 OXTr rs2254298	530 individuals	0.1287 0.0802
Domes et al., 2007	“Mind-reading”	OXT	30 participants	<0.006
Donaldson and Young, 2008	Social behavior	OXT AVP	Review article	–
Ebstein et al., 2009	Social behavior	OXTr AVPR1a	Review article	–
Ebstein et al., 2012	Social behavior	OXT, OXTr AVP, AVPR1a	Review article	–
Francis et al., 2016	Association with ASD	OXTr rs2268493 OXTr rs2254298-rs2268493 AVPR1a RS1-RS3 AVPR1b rs28632197 AVPR1b rs35369693	~200 families	0.05 0.026 <0.05 0.005 0.025
Furman et al., 2011	Amygdala Volume	OXTr rs2254298	51 participants	Left: 0.040 Right: 0.036
Gregory et al., 2009	OXTr deficiency in ASD	OXTr	119 probands	0.0389
Guastella and MacLeod, 2012	Review article	OXT	–	–
Guoynes et al., 2017	Intranasal OXT effects	OXT AVP	173 prairie voles	PVN: <0.05 SON: <0.01
Hammock and Young, 2002	Variation in social behavior	AVPR1a	Review article	–
Hammock and Young, 2006	Pair bonding – ASD	OXT AVPR1a	Review article	–
Haram et al., 2015	Emotional withdrawal	OXTr rs53576	1154 participants	0.007
Harony and Wagner, 2010	Social behavior and ASD	OXT AVP	Review article	–
Heinrichs and Domes, 2008	Social behavior	OXT AVP	Review article	–
Inoue et al., 2010	Amygdala volume	OXTr2254298	208 participants	0.004
Insel et al., 1999	Oxytocin, vasopressin and ASD	OXT AVP	Review article	–
Israel et al., 2008	Autism and altruism	OXTr AVPR1a RS3	203 participants	<0.05
Israel et al., 2009	Prosocial behavior	OXTr rs1042778	203 participants	0.001
Jacob et al., 2007	Association study	OXTr rs53576 OXTr rs2254298	57 trios	0.76 0.03
Kantojärvi et al., 2015	Association study	AVPR1a RS1	205 families	0.049
Kim et al., 2002	AVPR1a transmission disequlibrium in autism	AVPR1a RS1	115 trios	0.023
Lerer et al., 2008	Association study	OXTr rs2268494 OXTr rs1042778	152 subjects	0.0117 0.014
Lim et al., 2005	Animal model of autism	AVPR1a	Review article	–
Liu et al., 2010	Association study	OXTr rs53576 OXTr rs2254298	217 families	0.053 0.023
LoParo and Waldman, 2015	Association study	OXTr rs2254298	3941 individuals	0.0476
Marusak et al., 2015	Amygdala responses to social cues	OXTr rs2254298	55 participants	0.048
Meyer-Lindenberg and Tost, 2012	Amygdala activation and personality traits	AVPR1a RS1 AVPR1a RS3	228 participants	<0.03 –
Miller M. et al., 2013	Sex differences in autism	OXT AVP	75 participants	– Male: 0.03
Montag et al., 2017	Association study	OXTr rs2268498	817 students	<0.05
Moons et al., 2014	Emotional reaction to stress	OXTr rs53576 AVPR1a RS1	166 participants	Female: 0.39 Male: 0.012
Nyffeler et al., 2014	Association study	OXTr rs53576 OXTr rs2254298 OXTr rs2268494	253 participants	– 0.005 0.005
Parker et al., 2014	Social impairment prediction	OXTr rs53576 OXTr rs2254298	193 children	>0.05 0.0292
Peñagarikano, 2017	Animal model of autism	OXT	Review article	–
Poulin et al., 2012	Proscial behavior	OXTr rs53576 AVPR1a RS1 AVPR1a RS3	348 participants	<0.001 <0.001 –
Procyshyn et al., 2016	Association study	AVPR1a RS1 AVPR1a RS3	873 participants	– 0.0419
Rijlaarsdam et al., 2017	Association study	OXTr rs53576	746 children	<0.001
Rodrigues et al., 2009	Empathy and stress reactivity	OXTr rs53576	192 participants	>0.05
Sala et al., 2011	Animal model of autism	OXTr	Mice	<0.01
Saphire-Bernstein et al., 2011	Psychological resources	OXTr rs53576	261 participants	0.002
Schneider-Hassloff et al., 2016	G^*^E interaction and mentalizing	OXTr rs53576	195 participants	Amygdala voulme: *p* = 0.034
Shou et al., 2017	AVP pathways in ASD (fMRI study)	AVP	14 ASD children	*l*Amygd – *r*SMG: *p* = 0.02
Stoop, 2012	Neuromodulation	OXT AVP	Review article	–
Tansey et al., 2010	Association study	OXTr SNPs (18)	436 ASD subjects	>0.05
Tansey et al., 2011	Functionality of AVPR1a	AVPR1a rs11174815 AVPR1a RS1	177 families	0.008 0.036
Thompson et al., 2007	Prosocial effects	OXTr	Mice	<0.01
Tost et al., 2010	Prosocial effects and brain structure	OXTr rs53576	212 participants	Brain struct: 0.047 Prosociality: 0.05
Tseng et al., 2017	Breastfeeding and ASD	OXT	>1000 ASD children	0.002
Uzefovsky et al., 2015	Emotional and cognitive empathy	OXTr rs53576 AVPR1a RS3	367 participants	0.029 0.002
Veenema and Neumann, 2008	Regulation of complex social behavior	OXT AVP	Mice	<0.05
Veenema et al., 2010	Male aggression	AVP	Mice	<0.05
Verhallen et al., 2017	Face recognition	rs237887	370 participants	0.027
Walum et al., 2012	Social behavior	OXTr rs53576 OXTr rs2254298	40 participants	0.008
Wassink et al., 2004	Association study	AVPR1a	190 ASD subjects	0.02
Watanabe et al., 2017	Behavioral response in ASD	OXTr rs53576 OXTr rs2254298	38 ASD subjects	0.009 0.0009
Weisman et al., 2015	Cognitive empathy	OXTr rs53576	1463 participants	<0.05
Wermter et al., 2010	Association study	OXTr rs53576 OXTr rs2254298	100 ASD subjects	0.22 1.0
Wersinger et al., 2004	Social motivation	AVPR1b	Mice	<0.05
Winslow and Insel, 2004	Social recognition	AVPR1a AVPR1b	Review article	–
Wu et al., 2012	Empathy	OXTr rs2254298	101 participants	<0.05
Wu et al., 2015	Prosociality	AVPR1b rs28373064	256 participants	0.048
Wu et al., 2005	Association study	OXTr rs53576 OXTr rs2254298	195 ASD families	0.0101 0.0222
Yamasue, 2013	Autistic social behavior	OXTr rs53576 OXTr rs2254298	Review article	–
Yang et al., 2010a	Association study	AVPR1a RS1 AVPR1a RS3	148 trios	<0.001 <0.001
Yang et al., 2010b	Association study	AVPR1a rs7294536 AVPR1a rs10877969	151 trios	0.002 <0.001
Yang et al., 2017	Association study	AVPR1a RS1 AVPR1a RS3 AVPR1a rs7294536 AVPR1a rs10877969	212 families	– 0.02 0.039 <0.001
Yirmiya et al., 2006	Association study	AVPR1a RS1 AVPR1a RS3 AVR	116 families	0.004
Young, 1999	Social Behavior	OXTr AVPR1a	Mice	<0.05 <0.05
Yrigollen et al., 2008	Affiliative behavior and ASD	OXTr	151 families	<0.05
Zai et al., 2012	Child aggression	AVPR1b rs35369693	177 children	0.003
Zhang et al., 2017	Association with ASD	OXT AVP	Review article	–
Zimmermann et al., 2016	Social interaction and aggression	OXT	Zebrafish	<0.05

Oxytocin exerts its impact not only on the central nervous system, where it modulates functions that are relevant to the regulation of social behaviors, but also on autonomic responses, playing a role in motor, metabolic, sensory and visceral systems (Herman, 2012). It is largely expressed in the paraventricular nucleus (PVN) of the hypothalamus, an area where information related to cardiovascular and stress regulation converge and are relayed to downstream pathways, with subsequent effects on activities of the autonomic nervous system and hypothalamic-pituitary-adrenal (HPA) axis. It has been shown that from the PVN, oxytocin molecules maneuvre a preferential path to the amygdala, where it modulates emotional functions (Stoop, 2012).

### *OXTr* SNPs and autism

There is robust evidence to posit that oxytocin receptor gene plays a determining role in social behaviors, ranging from individual differences in non-clinical populations to ASD. Many studies have been conducted in the past two decades to better understand which polymorphisms have a bigger impact in the development of autism. A pioneer study by Horvath and colleagues found a significant correlation between A carriers in *OXTr* SNPs rs53576 and rs2254298 and autism (Horvath et al., 2001). Subsequently, other authors expanded our understanding of this relationship (LoParo and Waldman, 2015), finding a correlation between oxytocin receptor gene variants (rs53576, rs2254298 mainly) and autistic traits (Wu et al., 2005; Jacob et al., 2007; Lerer et al., 2008).

For *OXTr* rs53576, it has been demonstrated that A carriers, who are considered more vulnerable compared to G carriers, display reduced hypothalamic volume, that is shown to be related to susceptibility in ASD development (Liu et al., 2010; Tost et al., 2010; Jack et al., 2012; Wang et al., 2013). Many researchers have contributed studies which revealed the influence of this polymorphism on different social behavior. Specifically, there are evidences of correlations between rs53576 and prosocial disposition (Tost et al., 2010; Kogan et al., 2011; Yamasue, 2013), emotional withdrawal in psychotic disorders (Haram et al., 2015), negative affect (Lucht et al., 2009; Saphire-Bernstein et al., 2011), and reaction to stress and empathy (Rodrigues et al., 2009; Chen et al., 2011; Weisman et al., 2015; McDonald et al., 2016). Indeed, rs53576 has received much attention by researchers in the field of autism, with results suggesting that its involvement is not limited to ASD alone, but influences a wide spectrum of phenotypes related to general sociability and affiliative behaviors (LoParo and Waldman, 2015; Zhang et al., 2017). Generally, these results suggest that rs53576 is more related to sensitivity to social environment than to ASD itself (Tost et al., 2010; Jack et al., 2012; Wang et al., 2013).

Presently, studies on rs53576 have begun to investigate the role of epigenetic modification. For instance, Rijlaarsdam and colleagues have has recently discovered a significant interaction between *OXTr* methylation and child autistic traits, as well as social communication problems (Rijlaarsdam et al., 2017). Specifically, greater *OXTr* methylation was found to be correlated with social communication problems in rs53576 G-allele homozygotes, rather than A-allele carriers. Such findings encourage us to question the link that has been previously established between ASD susceptibility and the A-allele. This underscores the pertinence of accounting for epigenetic data in understanding the role of *OXTr* polymorphisms in ASD.

Another well-investigated polymorphism of *OXTr* is rs2254298, which is located in the third intron, with a guanine to adenine variation that is supposedly the “risk-inducing” form. As with rs53576, several studies attempted to understand the impact of rs2254298 on the development of ASD. Neuroimaging studies revealed a correlation between this variation and increased amygdala volume in general (Furman et al., 2011; Brüne, 2012) and when combined with environmental factors of early life stress (Marusak et al., 2015). Starting from the first genetic study on the etiology of autism in a Chinese population (Wu et al., 2005), others followed suit to find a significant correlation between rs2254298 and ASD diagnosis in Caucasian and Japanese samples (Jacob et al., 2007; Liu et al., 2010; LoParo and Waldman, 2015). Other findings centered on *OXTr* rs2254298 show that autistic traits are related to this polymorphism, but only when it is comprised in a haplotype (Lerer et al., 2008; Mosconi et al., 2009; Nyffeler et al., 2014; Francis et al., 2016). Besides autism, rs2254298 was also linked to social impairment in general (Parker et al., 2014), empathy (Wu et al., 2012) anxiety and depression (Mosconi et al., 2009; Thompson et al., 2011; Bittencourt Jacondino et al., 2014).

On the contrary, some authors found no significant correlation between *OXTr* rs2254298 and etiology of autism (Tansey et al., 2010; Campbell et al., 2011; Montag et al., 2017). An enlightening meta-analysis by Bakermans-Kranenburg and van Ijzendoorn emphasized the need to account for epigenetic mechanisms when examining SNPs like rs2254298 due to the lack of available research in the literature relating epigenetics not only to ASD development but to general social behavior (Bakermans-Kranenburg and van Ijzendoorn, 2014). Indeed, present data on genetic polymorphisms, up to the year 2014, fail to explain a considerable part of social behavior in humans. Another recent large study found no association between common variants of *OXTr* and *AVPr*, and social integration (Chang et al., 2014), although the authors suggested that the results could have been small. Additionally, the study was run on healthy non-clinical populations, not taking ASD diagnosis into account.

The results of the abovementioned studies pushed researchers to uncover the relationships between other *OXTr* variants in the promoter region and ASD in greater depth. Among these, rs2268493 has been found to correlate with ASD diagnosis (Yrigollen et al., 2008; Di Napoli et al., 2014), specifically with social withdrawal and repetitive behavior, both as an individual SNP and as part of a haplotype (Ebstein et al., 2009; Francis et al., 2016). There are also evidences of its relation with social cognitive performances in patients with schizophrenia (Davis et al., 2014) and in children diagnosed with ADHD (Ayaz et al., 2015); both disorders share features and symptoms with ASD. Campbell and colleagues found a statistically significant correlation of rs2268493 with autism but the significance was not retained after multiple comparison analysis (Campbell et al., 2011).

In the 3′-untranslated region of the *OXTr* gene, there is another SNP investigated in association with ASD. rs1042778 was found to be statistically correlated to ASD diagnosis in Caucasian and Jewish populations (Jacob et al., 2007; Lerer et al., 2008; Campbell et al., 2011), but the same result was not confirmed in the Japanese population (Liu et al., 2010). Furthermore, there are suggestions of its implication in aggression (Israel et al., 2009; Johansson et al., 2012; Malik et al., 2012) and prosocial behavior (Israel et al., 2009). With respect to rs13316193, there have been a few studies that found an association of this SNP, contained in a haplotype, with ASD (Ebstein et al., 2009; Liu et al., 2010). As an individual SNP, it has been associated with poor social abilities (Ebstein et al., 2012). Other SNPs have been tested in the last decade, but results are still few and contradictory (Apicella et al., 2010; Tansey et al., 2010), suggesting that these data need to be interpreted with caution and that more research is necessary. Other variants of *OXTr* that have been found to be related to autism and autistic traits are rs237897 (Lerer et al., 2008; Yrigollen et al., 2008; Wu et al., 2012; Kranz et al., 2016), rs237889 and rs2268494 (Lerer et al., 2008), rs237887 and rs237885 (Liu et al., 2010). Campbell and colleagues found a correlation between rs7632287 and multiple domains of ASD (Campbell et al., 2011) and childhood social problems such as pair-bonding between mother and child (Walum et al., 2012).

### AVPR SNPs and autism

Arginine-vasopressin is involved in regulatory mechanisms of social behavior like attachment, aggressive behavior and anxiety due to the activity of *AVP* receptors in the hypothalamic-pituitary-adrenal axis (Tansey et al., 2011). In a recent study, Shou and colleagues found that children with ASD, as compared to typically developing ones, showed a positive correlation between *AVP* levels in the plasma, increased volume in the left amygdala and left hippocampus, and decreased volume in the bilateral hypothalamus, suggesting a contribution of these structural and functional changes to the etiology of ASD (Shou et al., 2017). Studies about social behavior have shown the involvement of *AVPR1a* and *AVPR1b* gene polymorphisms (Avinun et al., 2011; Zai et al., 2012). Unlike oxytocin receptor gene, arginine-vasopressin receptor is composed by different encoder genes, such as *AVPR1a, AVPR1b* and *AVPR2*. In particular, two nucleotides located in the 5′ promoter region of *AVPr*; RS1 and RS3, have been the focus of research in this field, with evidence showing that these microsatellites modulate a wide range of social behaviors in both ASD and neurotypical populations (Kim et al., 2002; Wassink et al., 2004; Francis et al., 2016).

Arginine vasopressin receptor 1a (*AVPR1a*) is largely expressed throughout the brain (Kantojärvi et al., 2015), but also in the liver, kidneys, and vasculature. It is considered to be a crucial receptor for regulation of a set of social behaviors in humans (Ebstein et al., 2009, 2012; Francis et al., 2016), such as altruism and social integration (Israel et al., 2008; Knafo et al., 2008), prepulse inhibition (Levin et al., 2009), emotional and cognitive empathy (Israel et al., 2008; Knafo et al., 2008; Moons et al., 2014; Uzefovsky et al., 2015; Wang et al., 2016), processing and recognition of facial expressions (Israel et al., 2008; Knafo et al., 2008; Moons et al., 2014; Uzefovsky et al., 2015; Wang et al., 2016) and non-human mammals (Murakami et al., 2011; Freeman et al., 2014; Paré et al., 2016; Lesse et al., 2017). Even though vasopressin distribution patterns are quite similar across species, *AVPR1a* partitions differ both between and within species. In humans, vasopressin production occurs in the hypothalamus, bed nucleus of the stria terminalis, and the medial amygdala, while binding sites are located in the lateral septum, thalamus, basal amygdaloid nucleus, and brainstem (Meyer-Lindenberg et al., 2009; Zhang et al., 2017). Starting from the beginning of the century, many authors highlighted an association between variation at *AVPR1a* and neuropsychiatric phenotypes, with a wide focus on autism (Kim et al., 2002; Yirmiya et al., 2006; Tansey et al., 2011; Francis et al., 2016), parental behavior (Avinun et al., 2012) and social behavior in general (Yirmiya et al., 2006; Meyer-Lindenberg et al., 2009; Ebstein et al., 2012). Human *AVPR1a* promoter region contains two microsatellite repeats, which are highly polymorphic in the general population, and have been studied in association with individual variation in social behavior (Ebstein et al., 2012; Procyshyn et al., 2016): RS1, a (GATA)14 repeat sequence, and RS3, a complex (CT)4TT(CT)8(GT)24 repeat sequence (Thibonnier et al., 2000). Studies report an association between repeat number of these microsatellites and expression of *AVPR1a* in the hippocampus and the brain in general (Knafo et al., 2008). As a consequence, this can affect the modulation of social behavior on non-clinical autistic phenotypes (Carver and Harmon-Jones, 2009; Moons et al., 2014) and autistic traits (Israel et al., 2008; Procyshyn et al., 2016).

Specifically, results revealed an association between “short-alleles” of variant RS1 and a decrease in transcription of *AVPR1a* gene in the amygdala, leading to poorer prosocial behavior (Poulin et al., 2012; Kantojärvi et al., 2015; Yang et al., 2017) and higher amygdala activation during a facial recognition task (Meyer-Lindenberg et al., 2009). Kim et al. (2002) have been the first to conduct molecular genetic studies on *AVPR1a* and ASD, finding that one of the alleles of RS1 showed increased transmission in a family based study of 115 trios (mother, father, and child). More studies on the same microsatellite highlighted similar results (Wassink et al., 2004; Yirmiya et al., 2006; Yang et al., 2010a; Tansey et al., 2011; Kantojärvi et al., 2015). On the contrary, it is the “long-alleles” variant in RS3 that is associated with an increased in activity of the amygdala, which has been linked to social withdrawal. Studies show associations between this microsatellite and cognitive empathy and social skills in humans (Bosch and Neumann, 2008; Knafo et al., 2008; Harony and Wagner, 2010; Avinun et al., 2011; Uzefovsky et al., 2015), especially in the context of mother-infant play (Avinun et al., 2012). The “long-alleles” variant in RS3 has been linked to a risk of developing ASD too (Ebstein et al., 2009; Kantojärvi et al., 2015; Procyshyn et al., 2016). These results confirmed previous findings on animal models (Insel et al., 1994).

The studies reviewed show that *AVPR1a* modulation over brain functioning and ASD traits, with specific involvement in the domains of social communication, emotion and cognition, is often considered a consequence of the haplotype RS1-RS3 (Meyer-Lindenberg et al., 2009). Some authors have started investigating other SNPs belonging to the promoter region of *AVPR1a* gene to investigate if the etiology of autism might be modulated by a joint mechanism. An Irish study conducted in 2011 tagged four SNPs of the *AVPR1a* gene (rs3803107, rs1042615, rs3741865, rs11174815) but results showed that only rs11174815 was weakly correlated to autism (Tansey et al., 2011). Another study found a significant correlation of rs7294536, rs3759292, and rs10877969 on autistic phenotypes in trios in a Korean population (Yang et al., 2010b). Findings from these two studies suggest that the underlying actions of these receptors remain elusive, and that results may be influenced by specific cultural environments.

While the relationship between *AVPR1a* and ASD is relatively consistent, there is still need for further studies on the impact of AVPR1b on the outcome of autism. At the present, our knowledge is limited to the involvement of this receptor in the modulation of neuroendocrine response to stress (Muráni et al., 2010; Roper et al., 2011) and developmental disorders (Dempster et al., 2009; van West et al., 2009). Evidence about the role of *AVPR1b* in social and affiliative behaviors come from mice models (Wersinger et al., 2004; Caldwell et al., 2008, 2010; Pagani et al., 2015). There are few results that shed light on the involvement of *AVPR1b* in autistic traits in humans (Zai et al., 2012; Luppino et al., 2014; Wu et al., 2015), although it is possible to find evidence in literature that associates *AVPR1b* rs28632197 with comorbid conditions such as panic disorder (Keck et al., 2008). Exception being a recent report by Francis and colleagues on a correlation between *AVPR1b* rs35369693 and autism (Francis et al., 2016).

### *OXT, AVP* and differences between sexes

As already mentioned, ASD is well known to be sexually biased, occurring 4 times more in boys than girls. Studies on animal models, specifically in mammals, showed that the influence of *OXT* and *AVP* on brain activity and behavior is sexually-dimorphic, with stronger effects of oxytocin on females and of arginine-vasopressin in males. Hence, oxytocinergic and vasopressinergic systems may be related to the sex-biased occurrence of ASD.

Among the works discussed in the present review, there are few evidences showing sex-specific effects of *OXTr* and *AVPr* (and their variations), that enhanced the hypothesis of a sexually dimorphic modulation of autistic spectrum traits, and social behaviors in general, by these two neurotransmitters. Regarding *OXTr*, a study by Chen and colleagues found a significant sex-dependent effect in the interaction between rs2254298 and social functioning. Female A-carriers tended to show greater anxiety while men reported more autism-associated traits (Chen et al., 2011).

Furthermore, research by Chang and colleagues on social connectedness highlighted an opposite association between men and women with respect to rs4686302, and a direct correlation between rs53576 and women only, suggesting that sex can determine differential effects from the same variation (Chang et al., 2014). Other studies revealed sex-based differences in the amygdala, showing greater gray matter volume in male A-carriers for rs53576 (Tost et al., 2010) and a negative correlation with scores in reward tasks (Ebstein et al., 2012). The same amygdalial structural observation was also detected in homozygous G-carrier women for rs2254298 (Furman et al., 2011). Sex has also been shown to predict emotional empathy, with women scoring higher than men (Wu et al., 2012; Uzefovsky et al., 2015), although some studies did not find any differences (Rodrigues et al., 2009).

With regards to diagnosis of ASD, significant correlations were found between the Japanese male population and rs2254298 (Liu et al., 2010; Watanabe et al., 2017) and rs53576 (Watanabe et al., 2017). In the case of DNA methylation, as compared to healthy individuals, Gregory and colleagues found a significant sex-specific decrease in the expression of *OXTr* in males with autism, correlated to an increase in methylation at a specific site. Heterozygous deletion of the same *OXTr* site in mothers included in the sample did not result in diagnosis of ASD while deletion in males did (Gregory et al., 2009).

Switching to the vasopressin system, a few studies explored sex differences of *AVP, AVPr* and its variation on autism and social behaviors. Animal models showed that *AVP* in the central nervous system (CNS) activates brain regions (i.e., amygdala, hyppocampus) that coordinate specific-social behaviors, such as intermale aggression (Veenema et al., 2010); with regards to the receptors, it is possible to find in literature evidence that *AVPR1a* is necessary and sufficient for social recognition (Bielsky et al., 2005), and that deficits are worse in the male sample (Winslow and Insel, 2004). Furthermore, *AVP* in the CNS coordinates a range of different social behaviors. Rodents, specifically male samples of *AVPR1B* knockout mice, have also been used to elucidate mechanisms related to social dominance (Caldwell et al., 2010).

With regards to humans, prepulse inhibition (PPI) has been studied in both sexes, due to its involvement in social skills deficits. In 2009, two studies explicated the correlation between PPI and *AVPR1a* RS3, finding higher responses in long-carriers (Levin et al., 2009), which were even more evident in men (Ebstein et al., 2009). As for *AVPR1B*, rs28373064 was found to be more linked to prosociality in men compared to women (Wu et al., 2015) and this mechanism might be modulated by emotional empathy. A study conducted on children with ASD highlighted a strong association between *AVP* blood plasma levels and the connection between the left amygdala and left supermarginal gyrus, but only in boys (Shou et al., 2017). The amygdala is well-known to be a sexually-dimorphic major nucleus, receiving neurons from the hypothalamus that contains *AVP*, so this might shed light on mechanisms that contribute to the etiology of ASD.

With regards to separate analysis by sex, some incongruence is still present in literature (Knafo et al., 2008), underlining the need for lower variability and bigger sample sizes (Procyshyn et al., 2016). The included studies represent only a small portion of the evidences that underlie the sexually-dymorphic nature of *OXT* and *AVP* in the etiology of autism. Looking at these neurotransmitters together, a study by Miller and colleagues in an ASD population found higher levels of *OXT* in blood plasma in girls, and of *AVP* in boys; this difference was also reflected in a diverse association with autistic symptoms, especially with anxiety levels and repetitive behaviors (Miller M. et al., 2013). *OXTr* and *AVPr* genes have been found to interact with blood plasma levels of oxytocin and vasopressin in generating responses to emotional stress and these effects are displayed differently in men and women (Moons et al., 2014).

## Discussion

Since the early period of the animal kingdom, oxytocin has prevailed as a modulator of social affiliative behaviors across species of mammals, birds and amphibians alike (Gimpl and Fahrenholz, 2001; Donaldson and Young, 2008). Thereafter, came the emergence of a second integral neuropeptide which we have come to know as arginine-vasopressin, along with their respective receptors (Gimpl and Fahrenholz, 2001; Donaldson and Young, 2008). Like oxytocin, cells of the PVN and supraoptic nuclei of the hypothalamus express vasopressin too, conferring these two neuropeptides to a series of common features, starting from their parallel genetic structures, and eventually, similarities in how both these molecules exert their effects on the same neural structures in the central and autonomic nervous systems (Zhang et al., 2017). Being agonists, these two molecules are able to mutually bind to the other's receptors, influencing one another's functions. Although they share many common features, some differences between them do exist. For instance, *OXT* possesses a single receptor type, while *AVP* was found to have three different subtypes (*AVPR1a, AVPR1b, AVPR2)*. With regard to implication in social behaviors, *AVP* has been associated with pair-bond formation in monogamous species (Tickerhoof and Smith, 2017), while evidences in literature suggest that *OXT* is more broadly related to prosocial functioning. Furthermore, these two neuropeptides and their receptor variants exert different sex-dependent effects during physiological responses to stressors (Moons et al., 2014), suggesting an intriguing topic that still needs to be explored in-depth.

Beyond continuously developing knowledge about how *OXTr* and *AVPr* influence behaviors, researchers of today have devoted much attention to the roles these receptors play in the etiology of sex-biased neuropsychiatric disorders involving impairments on a social level, such as autism (Knapp et al., 2009). Indeed, numerous studies have been conducted to further uncover the effects of these neuropeptides in both sexes, revealing some sex-related differences on the “social behavior neural network” in clinical and non-clinical populations (Taylor et al., 2010; Dumais and Veenema, 2016; DiBenedictis et al., 2017). Directing resources toward the *OXT-AVP* system may potentially lead us to uncover critical biomarkers of ASD, allowing for earlier diagnosis, intervention and ultimately a more promising prognosis. An array of cutting-edge genetic tools, including high-throughput SNP sequencing, array-based copy number variant analysis, as well as genome-wide expression profiling, have surfaced in response to this need. These powerful molecular approaches, in combination with methods in neuroscience (i.e., imaging) and biochemistry (i.e., intranasal administration of *OXT*), pave the way for translational research that taps upon *OXT-AVP* pathways.

## Conclusion and future research

In this review, we have systematically examined the existing literature and have highlighted key SNPs of *OXTr* and *AVPr* that have been associated to ASD. The massive number of papers obtained from our search process illustrated how keen the scientific community is in endeavoring to converge disparate information from various fields (such as neurobiology, genetics, psychiatry, neuroscience, physiology, pharmacology, endocrinology, immunology) so as to generate new knowledge on ASD that can propel the development of novel preventive and intervention solutions.

*OXTr* and *AVPr* have been widely studied, being strongly related to social behavior. Specifically, RS1 and RS3, polymorphisms contained within the promoter region of *AVPR1*, and rs28632197 and rs35369693 SNPs of AVPR1b, have been found to be significantly linked to ASD. *OXTr* remains a key contributor as well, with rs53576 and rs225429 SNPs consistently shown to be associated with ASD. Controversy within the literature due to inconsistent findings have been discussed, including the varying effects of these polymorphisms on different ethnic groups, lack of agreement on which allele confers vulnerability for ASD, and even contention regarding the involvement of these SNPs in the first place. These discrepancies stem from inconsistent methodology, such as non-standardized inclusion criteria, sampling, data collection and analysis, and possibly requires systematic replication to be reconciled.

While much has been gleaned from studies investigating SNPs of *OXTr* and *AVPr*, the mechanisms behind how these polymorphisms contribute to ASD have yet to be fully elucidated. Three main suggestions for future studies can be generated from this systematic review. Firstly, given the male-biased nature of ASD, more emphasis should be placed on fully elucidating the differential mechanisms of *AVPr* and *OXTr* in males and females. Since sex-dependent differences in expression of these genes have been observed (Miller M. et al., 2013; Dumais and Veenema, 2016), this is a potential direction in which the field of genetics can take. However, the discrepancy in the prevalence between sexes in ASD in clinical populations might make this aim hard to achieve. Secondly, given the intricately similar characteristics and shared mechanisms of *AVP* and *OXT* on their receptor classes, additional studies should be conducted to uncover the interplay of these molecules at the genetic (i.e., polymorphisms of receptors), physiological (i.e., co-regulation of physiological pathways) and systemic levels (i.e., dual modulation of brain structures) in contributing to ASD development. Lastly, given the vastly contrasting outlook of DSM-5 from previous DSM manuals, with the former adopting the concept of “spectrum,” future meta-analyses should differentiate between the use of these manuals in ASD diagnosis.

More importantly, the story behind the development of autism has not been fully told until environmental influences have been investigated in conjunction with functional molecular analyses. It is largely plausible that *OXTr* and *AVPr* expression is more complex, regulated and fine-tuned at the cross-roads of cis-acting (i.e., SNPs) and trans-acting factors such as temporal and contextual variables (Carter, 2007). Even though replication studies have been conducted in the past few years, filling this gap in the literature, we have only uncovered the prodigious tip of the iceberg, and simultaneous effort across various research groups, from genetic through to the behavioral levels, is required to fully leverage upon the knowledge we have on *OXTr* and *AVPr* to generate effective diagnostic and therapeutic intervention for ASD.

## Author contributions

IC, AA, GE: Conceived, designed, and performed the review, interpreted literature, wrote the paper; and edited and reviewed the papers.

### Conflict of interest statement

The authors declare that the research was conducted in the absence of any commercial or financial relationships that could be construed as a potential conflict of interest.
